# Comparison of Pheromone Lures and Sticky Pad Color for Capturing *Tuta absoluta* (Lepidoptera: Gelechiidae)

**DOI:** 10.3390/insects16010094

**Published:** 2025-01-17

**Authors:** Muhammad Sadique, Muhammad Ishtiaq, Mirza Abdul Qayyum, Wafa A. H. Alkherb, Asim Abbasi, Muhammad Arshad, Unsar Naeem Ullah, Nazar Faried, Muhammad Irfan Akram, Nazih Y. Rebouh

**Affiliations:** 1 Institute of Plant Protection, Muhammad Nawaz Sharif-University of Agriculture, Multan 66000, Pakistan; 2 Department of Pest Warning and Quality Control of Pesticides, Government of Punjab, Lahore 54000, Pakistan; 3 Department of Biology, College of Science, Qassim University, P.O. Box 6666, Buraidah 51452, Saudi Arabia; 4 Department of Entomology, University of Agriculture, Faisalabad 38040, Pakistan; 5 Department of Horticulture, Muhammad Nawaz Sharif-University of Agriculture, Multan 66000, Pakistan; 6 Department of Entomology, Faculty of Agriculture and Environment, The Islamia University of Bahawalpur, Bahawalpur 63100, Pakistan; 7 Department of Environmental Management, Institute of Environmental Engineering, RUDN University, 6 Miklukho-Maklaya St., Moscow 117198, Russia

**Keywords:** sex pheromone, lure type, color sticky pad, Delta trap, IPM, mass trapping

## Abstract

Tomato leaf miner *Tuta absoluta* (Meyrick) (Lepidoptera: Gelechiidae) has gained the status of major pest globally. Integrated pest management (IPM) consists of different control methods. This field study was conducted to evaluate the influence of different pheromone-based traps to attract the male population and the potential of sticky pads of four different colors in capturing the adults in the absence of pheromone lures. The trials were conducted in Khyber Pakhtunkhwa (Pakistan) for two consecutive years, 2020 and 2021. The Delta trap with rubber septum type lure was significantly more attractive in both years, with mean number of capturing 76.0 and 86.17, as compared to polymer wax (32.83 and 28.2) and polymer vial (10.37 and 11.77), lures. The highest mean number of capturing of *T. absoluta* adults were noted as 17.93 and 21.73 with black color sticky trap without pheromone, whereas the lowest number of adults, i.e., 1.33 and 1.47, were captured during the first and second year of the study, respectively, with yellow color sticky traps. However, a significant difference was found in pheromone lures and among black, red, green, and yellow color sticky pads. Hence, Delta traps equipped with rubber septum pheromones lures and black colored sticky pads were more useful for the control of *T. absoluta* than any other colored sticky pads and pheromone lures alone. These findings could be helpful for the integrated management of *T. absoluta.*

## Simple Summary

Tomato leaf miner *Tuta absoluta* (Meyrick) (Lepidoptera: Gelechiidae) has emerged as a significant pest worldwide, necessitating effective pest management strategies. This study, conducted in Khyber Pakhtunkhwa, Pakistan, over two consecutive years (2020–2021), aimed to evaluate the efficacy of various pheromone-based traps and sticky pads in capturing male *T. absoluta* adults. The results demonstrated that Delta traps equipped with rubber septum pheromone lures were significantly more attractive, with mean captures of 76.0 and 86.17 adults in 2020 and 2021, compared to polymer wax (32.83 and 28.2) and polymer vial (10.37 and 11.77) lures. Additionally, among sticky traps without pheromone lures, black-colored pads proved most effective, capturing an average of 17.93 and 21.73 adults per year, while yellow pads were least effective, with captures of 1.33 and 1.47 adults. Significant differences were observed in both pheromone lure types and sticky pad colors. The findings suggest that Delta traps with rubber septum pheromones and black sticky pads offer a more effective approach for integrated management of *T. absoluta*.

## 1. Introduction

Tomato (*Solanum lycopersicum* L.) is an important and highly profitable vegetable crop which belongs to the genus *Solanum* and family *Solanaceae*. It is grown in open fields and under controlled conditions (greenhouses/foil tent) all over the globe [1,2]. In 2019, it was reported that the global production of tomato was 1.8 billion metric tons, which comes from an area of around 5.03 million hectares of farmlands [3,4]. Among these production statistics, Asia contributes 54.1% in global tomato production, followed by America (17.7%), Europe (15.9%), and Africa, which have a share of 11.9% [3]. The nutritional status of tomato is mainly attributed to its vitamins, minerals, and antioxidant components, which makes it a highly commercialized crop both in local and export markets [4]. In Pakistan, tomato is produced in all provinces in different seasons, particularly under tunnels during the off-season [5,6].

Biological invasions, especially of insect pests, have become a major challenge to modern agriculture due to their rapidly increasing spread either through vegetable and fruit trade or farm equipment. Moreover, the changing ecological and environmental conditions like temperature and humidity also favor the adaptions of a large number of insect pests in newly invaded areas [7,8]. Insect pests are the most severe constraints to tomato production, due to their direct physical damage and their role as facilitators of indirect losses, i.e., providing entry routes for various infectious pathogens like viruses, bacteria, fungi, and nematodes [4].

Tomato crops must survive in the presence of several insect pests, especially the tomato leaf miner, in all top tomato-producing countries [1]. *Tuta absoluta* (Meyrick) (Lepidoptera: Gelechiidae), commonly known as the South American tomato leaf miner, is a neotropical invasive pest, first found in Europe in 2006 in a tomato field grown under a controlled environment [9]. The pest has invaded Asian countries after European regions and countries located in the Mediterranean basin as well as African countries [10]. It was first reported in Pakistan in a tomato field cultivated under greenhouse conditions in 2018 [11]. It is an oligophagous pest and can damage many crops belong to the Solanaceae family [12,13], especially the tomato, on which it can cause yield losses ranging from 80 to 100% when no control measures are used [12].

Various biological parameters of *Tuta absoluta,* such as high reproductive potential, strong dispersal capability, multivoltinism, and multiple host ranges, have contributed towards its high damage potential to tomato crops throughout the world [14,15,16,17,18,19]. The larval feeding of *T. absoluta* causes substantial damage to all above-ground parts of tomato plants, including leaves, flowers, stem, and fruits [14]. The larvae also make galleries on leaves by feeding on the mesophyll tissues [20,21], causing significant yield losses and quality deterioration of the tomato fruits [22,23].

The presence and invasion of *T. absoluta* in Pakistan can increase the damage from pests both inside greenhouses as well as in open fields. This will indiscriminately increase the use of pesticides by net-house growers [24,25], as it is the most frequent method in Pakistan to counter insect pests [9,26]. However, *Tuta absoluta* is a challenging pest to manage with synthetic chemical insecticides due to the cryptic feeding habit of its larvae, which are protected inside the fruit or leaf mesophyll tissues [9]. Moreover, the frequent and injudicious application of pesticides and the shorter developmental duration of the pest favor the development of insecticide resistance in *T. absoluta* populations, thus further compromising the efficacy of chemical control tactics [17,27,28,29,30,31]. Insecticide use may also mess up integrated pest management programs previously used against tomato insect pests due to unsought effects on natural enemies and pollinators [32,33]. With the intention to reduce insecticide use on tomato crops, environmentally safe strategies like sex pheromones must be employed [34] as an eco-friendly alternative to synthetic insecticide.

Pheromone-based approaches like mating disruption and mass trapping have the potential to control this invasive pest [35,36]. Pest pressure of *T. absoluta* can be decreased effectively through mass trapping by removing enough males [37]. Mass trapping involves semiochemicals or some light to attract insects, combined with a physical trap or device to entrap them. The rates of insect catches vary with the traps used (water pan, adhesive surface, etc.). *T. absoluta* males are polygenic and on average mate 6.5 times in life. Therefore, to manage the pest population, the proportion of males must be removed sufficiently to reduce their chances of mating [37]. The number of adults captured in sex pheromone traps also helps in decision making and correct timing for launching management practices against *T. absoluta* [38,39]. Pheromone lures for tomato leaf miner of different types have been manufactured by many companies worldwide. They are promoted and tested by manufacturers and distributors or by experts themselves. The use of pheromone lures has reached more than 20 million lures based on the turnover of leading companies [37]. Color roll traps have been used in greenhouses [34] and in open fields [28] to capture *T. absoluta*. However, this study was performed with four different colors of sticky pads that could be used further in Delta traps with pheromone lures. The attractiveness and stability of three different types of pheromone lures were tested against *T. absoluta*. The aim of this study was to make the combination of Delta trap lures with the most effective sex pheromone and color sticky trap to enhance the trapping of *T. absoluta*.

## 2. Material and Methods

### 2.1. Experimental Site

This experiment was carried out in an open field in Pir Saddo Tehsil Takht Bhai District Mardan, Khyber Pakhtunkhwa, Pakistan (latitude 40.0″19′34° N and longitude 48.8″50′71° E: 310 m above sea level). A field of one hectare was selected for the experiments during the months of April and May for two subsequent years (2020 and 2021). Agronomic practices were carried out as per production technology and common farming practices of local farmers. No chemical spray was used before the experiment, whereas weeds were controlled mechanically as needed.

### 2.2. Trap and Experiment Design

A Delta trap was used for this experiment, made up of a transparent plastic sheet containing a sticky pad on the inner side. Sex pheromone lures/dispensers of different types, rubber septum, polymer wax, and polymer vial were stuck on the sticky pad almost in the center with three replications. Four traps, including control (without any pheromone dispenser), were placed in the tomato field at heights of about 50 cm to 100 cm above the ground depending upon the vegetation. Traps were installed a minimum of 10 m apart from each other [40]. Sticky pads of four different colors (red, green, yellow, and black) were used by hanging them at 50 to 100 cm height above the ground with the rope without any pheromone to check the color preference of *T. absoluta* adults with three replications. The experiment was conducted under RCBD with factorial arrangements. Color sticky pads were replaced every week as the efficacy of sticky gum decreased due to dust and other ecological factors.

### 2.3. Pheromone Lures

All types of emitting lures/dispensers contained the same standard dose of pheromone, 0.5 mg. A mixture of (3*E*,8*Z*,11*Z*)-3,8,11-tetradecatrienyl acetate and (3*E*,8*Z*)-3,8-tetradecadienyl acetate in a ratio of 90:10 was used in these lures [35,41] with different types of carrying materials, i.e., rubber septum, polymer vial, and polymer wax, provided by PK Biotec (Pvt.) Ltd. (Peshawar, Pakistan), to find the most effective one to capture *T. absoluta* in the long run. Pheromone lures were used once each year. However, sticky pads were replaced every week to keep them effective.

### 2.4. Data Collection

Data regarding male *T. absoluta* adults captured in each Delta trap and color sticky pad were recorded in the morning for five consecutive weeks on a weekly basis in both years. Moths/insects were visually counted directly in the field and their numbers were recorded.

### 2.5. Statistical Analysis

The data regarding adult captures of *T. absoluta* using sex pheromone and color sticky pad were analyzed by Statistics 8.1 statistical software (Analytical Software, Tallahassee, FL, USA). Means used for all pairwise comparisons were separated through Tukey HSD test at a 95% significance level [42].

## 3. Results

The results of analysis of variance (ANOVA) table for 2020 and 2021 displayed that the impact of treatments, i.e., sex pheromone dispensers and color sticky pads, were statistically highly significant (*p* ≤ 0.001) regarding adult captures of *T. absoluta.* In case of interaction effects during both years, interval × trap, interval × treatment, trap × treatment, and interval × trap × treatment were also highly significant (*p* ≤ 0.001) regarding adult captures of *T. absoluta* (Table 1).

The results of the experiment further showed that during 2020, the maximum mean number of adults were captured during first week (45.04), whereas the minimum number of adults (14.79) were captured during the last week. However, moth catches during the second (38.96) and third weeks (35.13) of 2020 were statistically non-significant from each other. The results further revealed that the mean number of *T. absoluta* adults captured during 2021 was also highly significant among the studied weeks. Maximum mean numbers of adults were captured during the first week (45.42) followed by the second week (40.92), third week (33.92), and fourth weeks (26.21), respectively, whereas the minimum number of adults were captured during the last week of the study interval (17.46) (Table 2).

Results regarding *T. absoluta* adults captured through Delta traps with the help of sex pheromone lures during both study years are presented in Table 3. Delta traps showed highly significant results regarding *T. absoluta* adult captures. The mean number of *T. absoluta* adults captured through rubber septum (76.00) was higher than those captured through polymer wax (32.83), polymer vial (10.37), and control (9.50) during the study period of 2020. However, the results of polymer vial and control were non-significant to each other regarding adult captures of *T. absoluta* during 2020 (Table 3).

Similarly, the mean number of *T. absoluta* adults captured during 2021 through rubber septum (86.17) was higher than those captured through polymer wax (28.20), polymer vial (11.77), and control (5.00). These results confirmed that rubber septum Delta traps performed significantly better than the rest of the traps regarding adult captures of *T. absoluta* during both study years (Table 3).

The efficacies of different colored sticky pads regarding *T. absoluta* adult captures are given in Table 4. The results demonstrated that all the tested colors varied significantly from each other regarding the capture of *Tuta absoluta* during both study years. During 2020, the highest numbers of adult moths of *T. absoluta* were attracted towards the black sticky pad (17.93). The black colored sticky pad was followed by red (15.20), green (8.47), and yellow (1.33). Similarly, Table 4 shows that for the year 2021, the highest number of adult moths of *T. absoluta* was attracted towards the black sticky pad (21.73) followed by red (14.93), green (11.27), and yellow (1.47) (Table 4).

Table 5 shows, for the years 2020 and 2021, the comparative efficacies of both treatments, i.e., sex pheromone dispensers and color sticky pads, regarding capture of *Tuta absoluta*. Both treatments varied significantly from each other regarding captures of *Tuta absoluta.* Sex pheromones were more efficient as compared to colored sticky pads alone when we studied them in comparison (Table 5).

Table 6 depicts the interaction of both treatments, i.e., sex pheromones and color sticky pads, with interval. The results of sex pheromone treatment with interval were highly significant during both study years, whereas color sticky pads were non-significant when studied with interval. The highest number of *T. absoluta* moths were attracted and captured through sex pheromone during the first week of both studied years (2020: 77.50 and 2021: 77.00) followed by the second, third, fourth, and fifth weeks. Sex pheromone dispenser efficiency decreased with the passage of time. A descending trend regarding adult captures was observed during the studied period for sex pheromone dispensers collectively (Table 6).

However, in the case of interval × color sticky pads, the collective results relating to adult captures were non-significant during both the studied years (Table 6). A stagnant trend of moth capturing was observed for the entire studied period. The results further revealed that the highest number of *T. absoluta* moths were captured through color sticky pads during the first week of both studied years (2020: 12.58 and 2021: 13.83). Later, the number of moth captures declined with each passing week during both studied years, except for the fifth week of 2021 (Table 6).

Table 7 discloses the comparison study of sex pheromone and color sticky pads collectively for the years 2020 and 2021. During 2020, the highest mean number of adults was captured through rubber septum (136.80) followed by polymer wax (57.20). Black colored sticky pads, polymer vial sex pheromone, and red colored sticky pads were statistically non-significant to each other. Yellow colored sticky pads were least effective in capturing *T. absoluta* adults (1.33). Similarly, for year 2021, the highest mean number of adults was captured through rubber septum (157.40) followed by polymer wax (45.13) (Table 7).

Table 8 shows, for the years 2020 and 2021, the interaction effect of interval × sex pheromone dispensers. During 2020, the results were highly significant among rubber septum dispenser and intervals. During the first week, the number of moth attracted was the highest (102.33) as compared to all other weeks. A similar trend was observed for the polymer wax sex pheromone dispenser, while for the polymer vial, the maximum number of adults was captured during the first week (16.00) and the lowest number was captured in the last week (2.33). However, the second, third, and fourth weeks were statistically non-significant to each other for the polymer vial during 2020. Moreover, in the control without any sex pheromone dispenser, the highest mean number of adults captured was during the first week (14.17), whereas for the rest of time they were statistically non-significant.

Table 8 shows, for the year 2021, significant results among rubber septum dispenser and intervals. However, the first and second weeks were non-significant to each other regarding moth captures. During these weeks, the number of moths attracted was the highest (1st: 116.67 and 2nd: 111.83) as compared to all other weeks. There were highly significant results for polymer wax. The maximum mean number was observed during the first week (42.00) followed by the second (34.83), third (32.50), fourth (21.33), and fifth (10.33) weeks. The results of the polymer vial dispenser were also significant among weeks. However, the first and the second week were non-significant to each other. Similarly, the third and the fourth week were also statistically non-significant to each other. Moreover, in the control without any sex pheromone dispenser, the highest mean number of adults captured was during the first week (14.17) and the fifth week (13.00), whereas during the second and fourth weeks, they were statistically non-significant.

## 4. Discussion

In the present study, two methods to capture the adults of the tomato leaf miner *T. absoluta* were used. Pheromone lure efficacy for the indication of pest incidence and mass trapping of *T. absoluta* has been reported by several studies [24,43,44,45]. Nowadays, chemical-based companies are involved in producing sex pheromone lures/dispensers that release pheromones at stable rates and for a longer time period to achieve better pest capture [46]. Moreover, in order to obtain stability and maximum efficiency for a longer time, manufacturers have increased the pheromone loads. The results of this comparative study show that the rubber septum capsule can attract more males as compared to polymer vials and polymer wax. A similar comparative study was conducted by Chermiti and Abbes [35], who reported that pheromone lures loaded with a higher dose of pheromone (0.8 mg) have more power to attract the *T. absoluta* males as compared to lures with a standard dose (0.5 mg). Similarly, Abbes and Chermiti [47] also reported that pheromone lures made by Russel IPM-UK have more attraction as compared to others, but more stability was shown by Koppert-type emitters.

The rubber septum showed more attraction, whereas stability was lower as compared to polymer wax. However, results concluded by Ferrara et al. [48] showed that a higher efficiency of pheromone lure was not directly proportional to a higher load of pheromone. However, release rate of pheromone and lure stability depend upon the amount of synthetic sex pheromone in the lures. It has been shown that more males are attracted toward the 1 μg doses as compared to 10 μg. All the pheromone lures in our study were loaded with a standard dose of 0.5 mg. Results were significant based on the difference in total number of adults captured in the whole experimental period. In both years, the rubber septum pheromone lure showed more efficiency in attracting the male adults, whereas the polymer wax pheromone lure showed comparatively more stability. The comparative effectiveness of pheromone lures remained the same in both study years; however, the effectiveness of traps/lures diminished during passing weeks. This decline in efficacy can be attributed to a number of factors such as trap types, pheromone lure substrate, lure longevity, trap height, and field positions [49,50,51,52].

Trap color had a substantial impact on number of adults captured [53]. It has been reported that *T. absoluta* can distinguish between black, blue, green, red, white, and yellow color traps [28,54]. The current results revealed that the yellow color was least attractive to *T. absoluta* as compared to black, red, and green. The study outcomes are in line with the findings of Roubos and Liburd [53] and Taha et al. [55], who found similar results when testing traps to capture grape root borer and *T. absoluta,* which are both nocturnal species. This might be due to light reflectance, which makes the color less noticeable at nighttime. However, in contrast to our results, Shiberu and Getu [56] reported that white and yellow traps were the most effective, whereas Polat and Tiryaki [28] reported that yellow color ferolite traps were the least effective as compared to other colors. Similar results were obtained in our study and also reported by Athanassiou et al. [57] against *Palpita unionalis* (Lepidoptera; Pyralidae).

For effective monitoring, a trap should capture a greater number of moths for the defined sampling period. The black color trap was the most effective in capturing the moths, followed by red and green. Similar results were reported by Polat and Tiryaki [28] for black and red, and Taha et al. [55] also reported similar results for red and green. In contrast to our results, Ferrara et al. [48] reported that a red color trap is the least effective, whereas Shiberu and Getu [56] reported that red and green were the least effective in capturing the *T. absoluta* adults.

The attraction of *T. absoluta* towards the color of sticky trap could improve the effectiveness of traps baited with pheromone. It would be helpful in early detection as well as obtaining the maximum number of adults that could be captured. The use of the black color would also prevent the capturing of pollinators which are attracted due to the flower’s color. Hassan et al. [34] stated that the use of yellow rolls in greenhouses to capture *T. absoluta* was not recommended because it could attract the pollinators/beneficial insects. However, we also found that yellow sticky traps were the least effective in capturing *T. absoluta* in current studies; therefore, it is recommended to use black instead of yellow in the field. Moreover, some parasitoids, *Dolichogenidea gelechiidivoris* and *Trichogramma* spp, which are natural enemies of *T. absoluta,* were also attracted to the commercial sex pheromone of *T. absoluta* [58]. Therefore, we recommend that future studies should test the impacts of these colors on the response of those parasitoids to the pheromones.

## 5. Conclusions

Trap efficiency, target specificity, and timely detection of pests are important aspects to take into consideration to develop monitoring and integrated pest management strategies. Sex pheromone baited traps can detect the pest presence accurately. All the tested lures attracted the *T. absoluta* males but exhibited different performance. Overall, in both years, the most attractive was the rubber septum as, compared to the poly vial and polymer wax. This study showed that sticky trap colors strongly affect the *T. absoluta* response. Colored sticky traps and sex pheromone lures are useful tools for the monitoring and detection of the occurrence and abundance of *T. absoluta*. In particular, the combination of the black color sticky trap baited with the rubber septum sex pheromone lure could be more effective than the other sticky trap colors and pheromone lures. These findings will be helpful in monitoring and developing integrated pest management strategies for *T. absoluta*, but this technique will be more effective when implemented by all growers in a region simultaneously. Further research is recommended to understand the effects of colored traps on non-target insects.

## Figures and Tables

**Table 1:** Analysis of variance (ANOVA) for evaluation of sex pheromone and color sticky pads against *Tuta absoluta* during 2020 and 2021.

	2020					2021				
Sources	DF	SS	MS	F	P	DF	SS	MS	F	P
Replication	2	376	188.0			2	183	91.5		
Interval	4	13,192	3298.0	112.04	0.0000	4	12,123	3030.7	184.45	0.0000
Trap	3	87,325	29,108.0	988.85	0.0000	3	122,532	40,844.0	2485.73	0.0000
Treatment	1	55,169	55,169.4	1874.20	0.0000	1	50,103	50,102.5	3049.19	0.0000
Interval × Trap	12	8387	698.9	23.74	0.0000	12	13,392	1116.0	67.92	0.0000
Interval × Treatment	4	10,532	2633.1	89.45	0.0000	4	11,493	2873.3	174.87	0.0000
Trap × Treatment	3	77,260	25,753.4	874.88	0.0000	3	114,080	38,026.6	2314.26	0.0000
Interval × Trap × Treatment	12	10,175	847.9	28.81	0.0000	12	13,979	1164.9	70.90	0.0000
Error	78	2296	29.4			78	1282	16.4		
Total	119	264,713				119	339,166			

**Table 2:** Weekly comparison of mean number of trapped *Tuta absoluta* adults in 2020 and 2021.

Interval	Mean No. of Capturing	
	2020	2021
1st week	45.04 A	45.42 A
2nd week	38.96 B	40.92 B
3rd week	35.13 B	33.92 C
4th week	26.96 C	26.21 D
5th week	14.79 D	17.46 E

Mean numbers with different letters are statistically significant from each other; *p* ≤ 0.05. Mean numbers with the same letters are statistically non-significant; *p* > 0.05 (HSD test).

**Table 3:** Comparison of sex pheromone dispensers in trapping *Tuta absoluta* adults in 2020 and 2021.

**Sex Pheromone Dispenser Types**	**Mean No. of Capturing**	
	**2020**	**2021**
Rubber septum	76.00 A	86.17 A
Polymer wax	32.83 B	28.20 B
Polymer vial	10.37 C	11.77 C
Control	9.50 C	5.00 D

Mean numbers with different letters are statistically significant from each other; *p* ≤ 0.05. Mean numbers with the same letters are statistically non-significant; *p* > 0.05 (HSD test).

**Table 4:** Comparison of color sticky pads in trapping *Tuta absoluta* adults in 2020 and 2021.

Color Sticky Pad	Mean No. of Capturing	
	2020	2021
Black	17.93 A	21.73 A
Red	15.20 B	14.93 B
Green	8.47 C	11.27 BC
Yellow	1.33 D	1.47 C

Mean numbers with different letters are statistically significant from each other; *p* ≤ 0.05 (HSD test).

**Table 5:** Evaluation of sex pheromone dispensers and color sticky pads in trapping *Tuta absoluta* adults in 2020 and 2021.

Sex Pheromone × Color Sticky Pad	Mean No. of Capturing	
	2020	2021
Sex pheromone	53.62 A	53.22 A
Color sticky pad	10.73 B	12.35 B

Mean numbers with different letters are statistically significant from each other; *p* ≤ 0.05 (HSD test).

**Table 6:** Comparison of mean number of trapped *Tuta absoluta* adults for interval × treatments in 2020 and 2021.

Interval	Treatment	Mean No. of Capturing	
		2020	2021
1st week	Sex pheromone	77.50 A	77.00 A
2nd week	Sex pheromone	67.08 B	69.83 B
3rd week	Sex pheromone	59.00 C	56.00 C
4th week	Sex pheromone	44.00 D	41.17 D
5th week	Sex pheromone	20.50 E	22.08 E
1st week	Color sticky pad	12.58 F	13.83 F
2nd week	Color sticky pad	10.83 F	12.00 F
3rd week	Color sticky pad	11.25 F	12.00 F
4th week	Color sticky pad	9.92 F	11.25 F
5th week	Color sticky pad	9.08 F	12.83 F

Mean numbers with different letters are statistically significant from each other; *p* ≤ 0.05. Mean numbers with the same letters are statistically non-significant; *p* > 0.05 (HSD test).

**Table 7:** Comparison of mean number of trapped *Tuta absoluta* adults for trap type × treatments in 2020 and 2021.

Trap Type	Treatment	Mean No. of Capturing	
		2020	2021
Rubber septum	Sex pheromone	136.80 A	157.40 A
Polymer wax	Sex pheromone	57.20 B	45.13 B
Polymer vial	Sex pheromone	17.67 C	8.53 E
Control	Sex pheromone	2.20 DE	1.80 F
Red	Color sticky pad	15.20 C	14.93 D
Black	Color sticky pad	17.93 C	21.73 C
Green	Color sticky pad	8.47 D	11.27 DE
Yellow	Color sticky pad	1.33 E	1.47 F

Mean numbers with different letters are statistically significant from each other; *p* ≤ 0.05. Mean numbers with the same letters are statistically non-significant; *p* > 0.05 (HSD test).

**Table 8:** Comparison of mean number of trapped *Tuta absoluta* adults for interval × trap type in 2020 and 2021.

Interval	Trap Type	Mean No. of Capturing	
		2020	2021
1st week	Rubber septum	102.33 A	116.67 A
2nd week	Rubber septum	92.17 AB	111.83 A
3rd week	Rubber septum	83.83 B	87.67 B
4th week	Rubber septum	66.00 C	70.33 C
5th week	Rubber septum	35.67 EF	44.33 D
1st week	Polymer wax	47.67 D	42.00 DE
2nd week	Polymer wax	41.17 DE	34.83 EF
3rd week	Polymer wax	35.67 EF	32.50 F
4th week	Polymer wax	26.33 FG	21.33 G
5th week	Polymer wax	13.33 HI	10.33 HIJ
1st week	Polymer vial	16.00 GH	8.83 HIJ
2nd week	Polymer vial	12.33 HI	6.50 HIJ
3rd week	Polymer vial	10.17 HI	4.33 IJ
4th week	Polymer vial	6.67 HI	3.17 IJ
5th week	Polymer vial	2.33 I	2.17 J
1st week	Control	14.17 H	14.17 GH
2nd week	Control	10.17 HI	10.50 HIJ
3rd week	Control	10.87 HI	11.17 HI
4th week	Control	8.83 HI	10.00 HIJ
5th week	Control	7.83 HI	13.00 GH

Mean numbers with different letters are statistically significant from each other; *p* ≤ 0.05. Mean numbers with the same letters are statistically non-significant; *p* > 0.05 (HSD test).

## Data Availability

The data used and analyzed during this research work can be requested from corresponding authors on reasonable requests.
